# Targeting the WEE1 kinase as a molecular targeted therapy for gastric cancer

**DOI:** 10.18632/oncotarget.10231

**Published:** 2016-06-23

**Authors:** Hye-Young Kim, Yunhee Cho, HyeokGu Kang, Ye-Seal Yim, Seok-Jun Kim, Jaewhan Song, Kyung-Hee Chun

**Affiliations:** ^1^ Department of Biochemistry & Molecular Biology, Yonsei University College of Medicine, Seodaemun-gu, Seoul 03722, Korea; ^2^ Department of Biochemistry, College of Life Science and Biotechnology, Seodaemun-gu, Seoul 03722, Korea; ^3^ Brain Korea 21 PlusProject for Medical Science, Yonsei University, Seodaemun-gu, Seoul 03722, Korea

**Keywords:** WEE1, AZD1775 (MK-1775), 5-FU, Paclitaxel, gastric cancer

## Abstract

Wee1 is a member of the Serine/Threonine protein kinase family and is a key regulator of cell cycle progression. It has been known that WEE1 is highly expressed and has oncogenic functions in various cancers, but it is not yet studied in gastric cancers. In this study, we investigated the oncogenic role and therapeutic potency of targeting WEE1 in gastric cancer. At first, higher expression levels of WEE1 with lower survival probability were determined in stage 4 gastric cancer patients or male patients with accompanied lymph node metastasis. To determine the function of WEE1 in gastric cancer cells, we determined that WEE1 ablation decreased the proliferation, migration, and invasion, while overexpression of WEE1 increased these effects in gastric cancer cells. We also validated the clinical application of WEE1 targeting by a small molecule, AZD1775 (MK-1775), which is a WEE1 specific inhibitor undergoing clinical trials. AZD1775 significantly inhibited cell proliferation and induced apoptosis and cell cycle arrest in gastric cancer cells, which was more effective in WEE1 high-expressing gastric cancer cells. Moreover, we performed combination treatments with AZD1775 and anti-cancer agents, 5- fluorouracil or Paclitaxel in gastric cancer cells and in gastric cancer orthotopic-transplanted mice to maximize the therapeutic effect and safety of AZD1775. The combination treatments dramatically inhibited the proliferation of gastric cancer cells and tumor burdens in stomach orthotopic-transplanted mice. Taken together, we propose that WEE1 is over-expressed and could enhance gastric cancer cell proliferation and metastasis. Therefore, we suggest that WEE1 is a potent target for gastric cancer therapy.

## INTRODUCTION

Gastric cancer is the fourth leading cause of cancer and the second leading cause of cancer-related death worldwide [1]. Usually, gastric cancer occurs in the mucosal layer of the stomach wall, and it can be removed with surgery. However, in cases of metastasis to other organs, surgical methods are not suitable for the treatment of metastasis. This is why anti-cancer chemotherapy (i.e., anticancer agents, anti-cancer injection or drug treatment) is needed to treat cancer patients. Also, anti-cancer specific drug development and molecular level reaction mechanism research is needed to develop effective anti-cancer treatments [2].

Many anti-cancer agents induce cell-cycle associated-DNA damage. Cell-cycle checkpoints allows enough time for the maintenance of genomic integrity in response to DNA damage, and serve to stop the progression of the cell cycle [3]. Normal cells repair damaged DNA during G1-arrest, however cancer cells often have deficient G1-arrest and largely depend on G2-arrest. Thus, cancer cells have increased DNA damage at the G2-checkpoint compared to normal cells [4]. The molecular switch for the G2–M transition is held by WEE1 and is pushed forward by CDC25 [5]. WEE1 is a nuclear kinase that belongs to a family of protein kinases involved in terminal phosphorylation and are functional activation during the S/G2 phase of the cell cycle [6, 7]. WEE1 was first discovered in a cell division cycle mutant – *wee1* – in fission yeast (*Schizosaccharomyces pombe*) [8]. Deletion of WEE1 in fission yeast was characterized by a smaller cell size. This phenotype has been attributed to the function of WEE1 in regulating the inactivity of the cyclin-dependent kinase Cdc2 (Cdc28 in budding yeast and CDK1 in humans) in the Cdc2/CyclinB complex [9]. WEE1 is associated with response to chromatin synthesis and response to DNA damage. Once DNA damage has occurred in the cell, WEE1 inhibits the cell cycle in the S/G2 phase through CDK1 Tyr15 phosphorylation [10]. Recently, WEE1 was shown to directly phosphorylate the mammalian core histone H2B at tyrosine 37. Inhibition of WEE1 kinase activity, such as with a WEE1 inhibitor or through suppression of its expression by RNA interference, abrogated H2B Y37-phosphorylation with a concurrent increase in histone transcription [11]. Therefore, WEE1 has a dual role in S-phase regulation and histone synthesis, such as a key regulator of chromatin integrity. WEE1 specific inhibitors cause mitotic infidelity, chromosome loss, and apoptosis, and these effects are referred to as a mitotic catastrophe [12].

Overexpression of WEE1 has been reported in several cancers, such as malignant melanoma, breast cancer, osteosarcoma and glioma [13–16]. Among of them, malignant melanoma and high-grade glioma patients with WEE1 high-expression showed to correlate with malignancy [13, 16]. However, the study of WEE1 in gastric cancer cells has not been reported yet. Moreover, WEE1 inhibitors are undergoing clinical trials [17]. AZD1775, also known as MK-1775, is a pyrazolo-pyrimidine derivative that is a selective inhibitor of the WEE1 kinase with effective checkpoint inhibitory activation [18]. AZD1775 is a highly selective, potent, ATP competitive, small molecule inhibitor of Wee1 kinase [19]. Toxicity studies from a Phase I trial with AZD1775 suggest that AZD1775 could be safely combined with a variety of chemotherapy agents to treat solid tumors [20]. Preclinical studies have demonstrated potent chemo-sensitizing activities when AZD1775 is combined with S-phase toxins, such as DNA cross-linking agents, nucleoside analogs or inhibitors of DNA metabolism, or topoisomerase poisons. [21]. Therefore, we started this study to identify the role of WEE1 in proliferation and motility in gastric cancer, and we also determined the potential for making WEE1 a therapeutic target in gastric cancer.

## RESULTS

### High expression of WEE1 is associated with poor prognosis in male gastric cancer patients with lymph node metastasis

**Figure 1:**
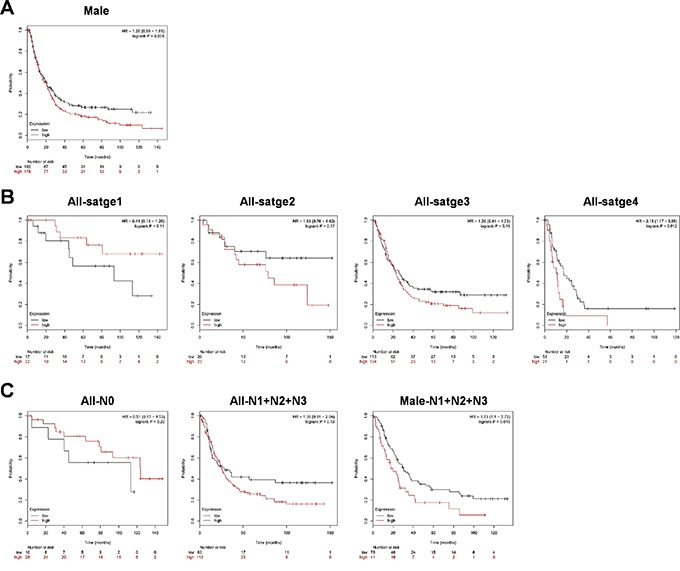
Survival rate of WEE1 expression is detected in gastric cancer patients using the Kaplan-Meier Plotter **A.** Overall survival rates of male gastric cancer patients (n=360, right panel, p=0.056). **B.** Overall survival rates for each patient stage of gastric cancer. Stage1 (All-stage1, p=n.s) n=39, stage2 (All-stage 2, p=n.s) n=49, stage3 (All-stage 3, p=n.s) n=217, and stage4 (All-stage 4, p=0.0012) n=74. **C.** Overall survival rates for each patient stage of gastric cancer with lymph node metastasis. Lymph node metastasis stage0 (All-N0, p=n.s) n=38, lymph node metastasis stage1-3 (All-N1+N2+N3, p=n.s) n=175, lymph node metastasis stage1-3 of males (Male-N1+N2+N3, p=0.016) n=119.

To assess the prognostic value of WEE1 expression in gastric cancer patients, the association between WEE1 expression and survival was analyzed using Kaplan-Meier analysis. Overall survival rates for the gastric cancer patients were not significant based on expression of WEE1 (data not shown). However, male gastric cancer patients showed high-expression of WEE1 with poor survival probability (Figure 1A). We checked the overall survival rates for each stage of gastric cancer; gastric cancer stage 1, stage 2, and stage 3 did not significantly correlate with WEE1 expression, whereas the prognosis for stage 4 of gastric cancer patients showed very poor and low survival probability with higher WEE1 expression (Figure 1B). Lymph node metastasis stage 0 and stage 1-3 did not significantly correlate with WEE1 expression. However, male gastric cancer patients with lymph node metastasis stage 1-3 showed a correlation with poor prognosis and higher WEE1 expression (Figure 1C). These results suggest that the prognosis of male gastric cancer patients was affected by the expression of WEE1 with poor prognosis. Also, massive lymph node metastasis of male gastric cancer patients was associated with high expression of WEE1.

### Ablation of WEE1 decreased the viability, invasion, and migration of gastric cancer cells

**Figure 2:**
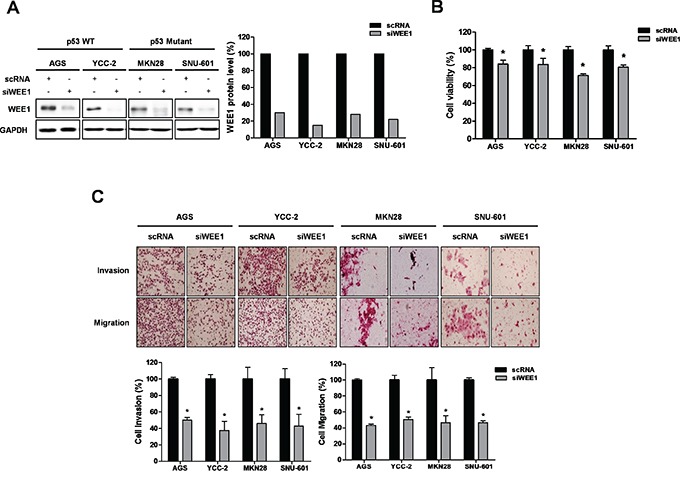
Down-regulation of WEE1 reduces cell viability and proliferation AGS, YCC-2, MKN28, and SNU-601 were transfected with scRNA and WEE1 siRNA (siWEE1). **A.** WEE1 protein expression was detected by Western blot. GAPDH was used as a loading control. The density of WEE1 expression by western blot analysis was measured by densitometry and calculated and presented as a left graph. **B.** WST assays were performed to detect cell viability in transfected scRNA or siWEE1 cells. **C.** Trans filter well assays with matrigel were performed to detect the invasion activity of transfected siWEE1 cells. Trans filter well assays with collagen were used to detect the migration activity of transfected siWEE1 cells. Also, transfected siWEE1 cells had reduced cell invasion and migration. Data is represented as mean ± SD (n=3). The significant differences are indicated by asterisk (* p<0.05), p values were calculated using student t tests.

We determined the expression levels of WEE1 in twelve gastric cancer cell lines (AGS, YCC-2, MKN28, KATO III, SNU-1, SNU-5, SNU-16, SNU-216, SNU-601, SNU-638, SNU-668, and SNU-719) (Figure S1). We selected WEE1 high-expressing cells, such as AGS (p53 wild type), YCC-2 (p53 wild type), MKN28 (p53 I251L mutant), and SNU-601 (p53 R273H mutant). To investigate the effect of WEE1 silencing on cell viability in gastric cancer cells, AGS, YCC-2, MKN28, and SNU-601 were transfected with three different sequences WEE1 siRNAfor 2 days (Figure S2), and #1 was selected and used this study. We detected a decrease in WEE1 expression (Figure 2A) as well as inhibited cell viability in WEE1 siRNA-transfected cells (Figure 2B). We also determined the effect of WEE1 silencing on cell invasion and migration (Figure 2C). Ablation of WEE1 significantly reduced invasion and migration in gastric cancer cells. These results suggest that ablation of WEE1 inhibited cell viability, invasion, and migration in gastric cancer cells.

### Overexpression of WEE1 increased the viability, invasion, and migration of gastric cancer cells

**Figure 3:**
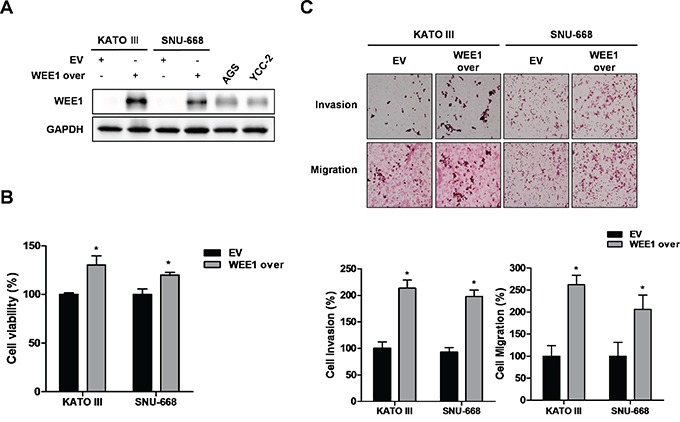
Over-expression of WEE1 rescues cell proliferation, invasion, and migration Over-expression of WEE1 in KATO III and SNU668 cells was achieved by transfection with an empty vector (EV) or WEE1 over-expression vector (WEE1 over). **A.** Expression of WEE1 protein was detected by Western blot analysis. GAPDH was used as a loading control. **B.** WST assays were performed to detect cell viability in transfected EV or WEE1 over-expressed cells. **C.** Trans filter well assays were performed to detect the invasion ability of the transfected EV or WEE1 over-expressed cells. Trans filter well assays with collagen were used to detect the migration activity of transfected EV or WEE1 over-expressed cells. Also, transfected WEE1 over-expression cells showed increased cell invasion and migration. Data is represented as mean ± SD (n=3). Significant differences are indicated by asterisk (* p<0.05), p value were calculated using student t tests.

For the gain-of-function study, we selected the WEE1 low-expressing cells, such as KATO III and SNU-668, and investigated the effect of WEE1 over-expression on cell viability in gastric cancer cells. After KATO III and SNU-668 cells were transfected with the empty vector (EV) and the WEE1 over-expression vector, we detected an increase in WEE1 expression (Figure 3A). Over-expression of WEE1 significantly increased cell viability (Figure 3B). We also determined the effect of WEE1 on the invasion and migration of gastric cancer cells. Over-expression of WEE1 increased gastric cancer cell invasion and migration (Figure 3C). These results suggest that over-expression of WEE1 enhanced the viability, invasion, and migration of gastric cancer cells.

### AZD1775, a WEE1 inhibitor, inhibited the viability of gastric cancer cells

**Figure 4:**
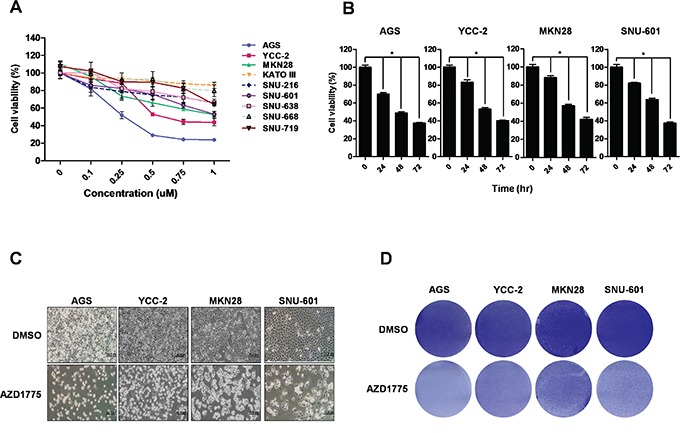
Treatment with WEE1 inhibitor (AZD1775) in gastric cancer cell lines reduces cell viability in a dose-dependent and time-dependent manner **A.** WST assays were performed to detect the cell viability by a dose-dependent AZD1775 treatment for 48 hrs in nine gastric cancer cell lines. **B.** WST assays were performed to detect the cell viability of treated cells with time-dependent AZD1775 treatment in AGS, YCC-2, MKN28, and SNU-601 cells. **C.** and **D.** After AZD1775 treatment for 48 hrs, morphology and density of four gastric cancer cells were assessed by microscope and crystal violet staining, respectively. Scale bar represents 20 μm.

We treated nine human gastric cancer cell lines (AGS, YCC-2, MKN28, KATO III, SNU-216, SNU-601, SNU-638, SNU-668, and SNU-719) with AZD1775 and determined cell viability in a dose-dependent manner for 48 hrs (Figure 4A). Seven cell lines (AGS, YCC-2, MKN28, SNU-216, SNU-638, SNU-601, and SNU-719 cells) showed decreased cell viability by dependent manner with concentration of AZD1775, whereas KATO III and SNU-668 cells showed resistance with AZD1775. This sensitivity corresponded with WEE1 expression in gastric cancer cells; WEE1 high-expressing cells were more sensitive than WEE1 low-expressing cells. Furthermore, we treated WEE1 high-expressing cells (AGS, YCC-2, MKN28, and SNU-601 cells) with AZD1775 and determined cell viability in a time-dependent manner (Figure 4B-4D). We analyzed the IC_50_ by WST assay (data not shown). And then, we treated with IC_50_amounts; AGS and YCC-2 cells, p53 wild type cells, were treated with 0.5 μM of AZD1775, and MKN28 and SNU-601, p53 mutant cells, were treated with 1 μM of AZD1775. Even though IC_50_ is dependent of p53 status, all of the cells showed decreased cell viability in a time-dependent manner (Figure 4B). Decreases in cell number were also detected by microscope (Figure 4C) and by staining with crystal violet (Figure 4D). These data suggest that AZD1775 significantly reduced cell viability in gastric cancer cells.

### AZD1775 induced cell cycle arrest and apoptosis in gastric cancer cells

**Figure 5:**
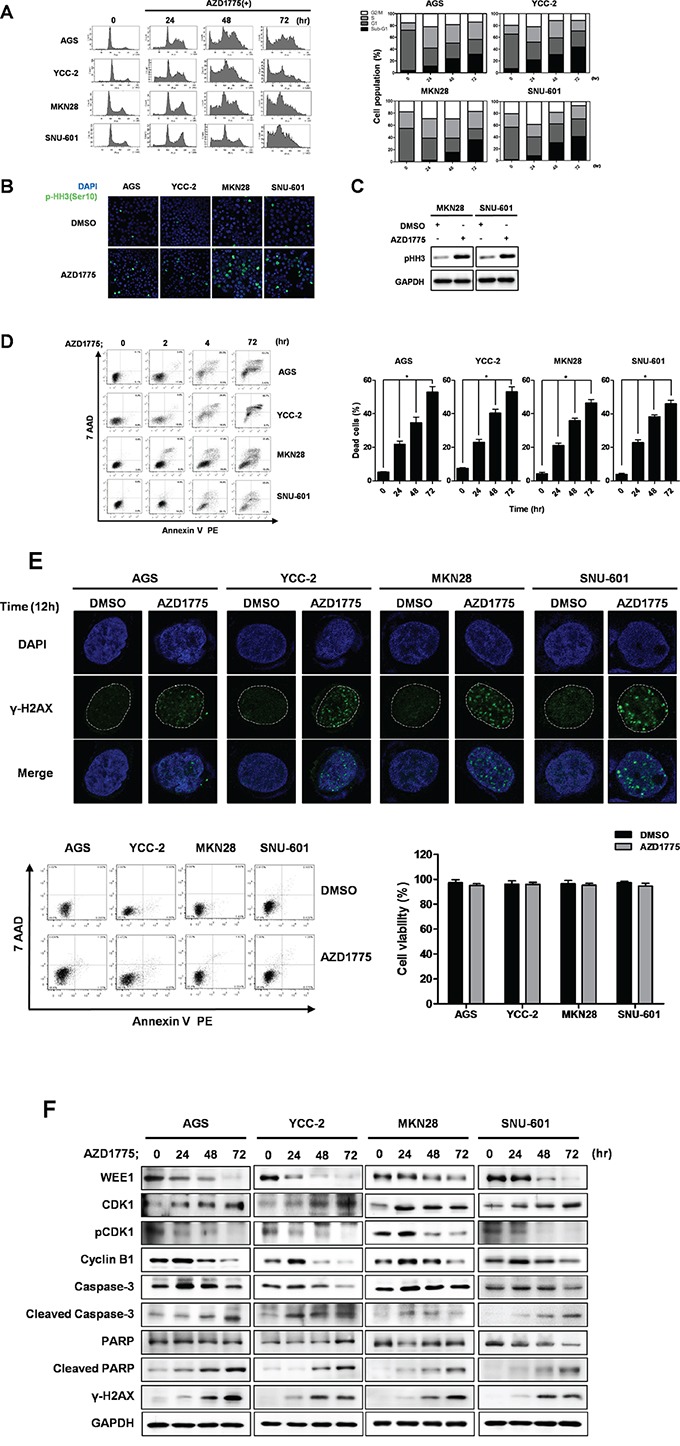
AZD1775 induces cell cycle arrest and apoptotic cell death in gastric cancer cells **A.** Cell cycles of AGS, YCC-2, MKN28, and SNU-601 cells were detected by FACS analysis using PI staining. Quantitative data are presented graphically. AZD1775 treated cells at the indicated times and concentrations. After AZD1775 treatment for 24 hrs, Phosphorylated Histone H3 (Ser 10) (pHH3) was detected by immunofluorescence staining **B.** western blot analysis **C. D.** Apoptosis rates of AGS, YCC-2, MKN28, and SNU-601 cells were detected by FACS analysis using annexin-V staining. Quantitative data are presented graphically. AZD1775 treatments were for the indicated times and concentrations. Data is represented as mean ± SD (n=3). Significant differences are indicated by asterisk (* p<0.05), p values calculated using ANOVA. **E.** After AZD1775 treatment for 12 hrs, γH2AX was detected by immunofluorescence staining. Apoptosis and cell viability were detected and presented lower panel. **F.** Apoptosis related proteins were detected by Western blot. AGS, YCC-2, MKN28, and SNU-601 cells were treated with AZD1775 for the indicated times. GAPDH was used as a loading control.

We examined the cell cycle population and monitored apoptosis induction after treatment with 0.5 μM of AZD1775 in AGS and YCC-2cells, and with 1 μM of AZD1775 in MKN28 and SNU-601 cells (Figure 5). Treatment with AZD1775 induced cell cycle arrest starting at 24 hr sand up to 72 hrs in a time-dependent manner (Figure 5A). Interestingly, p53 wild type AGS and YCC-2 cells were induced G1/S phase arrest and subsequently the sub-G1 population was increased after 48 hrs. On the other hand, p53 mutant MKN28 and SNU-601 cells were induced G2/M phase arrest and the sub-G1 population was increased after 48 hrs (Figure 5A). After treatment with AZD1775 for 24 hrs, we detected increased phosphorylated-Histone H3-stained cells in p53 mutant MKN28 and SNU-601 cells. We also detected increased phosphorylation of Histone H3 by western blot analysis in these two cells (Figures 5B and 5C).

Next, we detected apoptosis induction using annexin V staining and FACS analysis (Figure 5D). There was a time-dependent increase in annexin V binding cells after AZD1775 treatment. Untreated gastric cancer cells exerted low background staining with annexin V (<10%), whereas after incubation with AZD1775 for 24 hrs, gastric cancer cells showed positive annexin V staining and negative 7-AAD staining, indicating that they were undergoing early apoptosis. After 48 hrs, more 20% of cells were annexin V-positive and 7-AAD–negative stained. However, more 40% of the cells were positive stained with both annexin V and 7-AAD, indicating that they were undergoing late apoptosis after 72 hrs of AZD1775 treatment.

Furthermore, we determined the DNA damage effect of AZD1775 in gastric cancer cells (Figure 5E). To avoid coincidence with DAN fragmentation by apoptosis, we treated for 12 hrs and detected the expression γ-H2AX and formation of Foci. After AZD1775 treatment, significant expression γ-H2AX and formation of Foci were detected in all these cells without apoptosis or cell death induction.

We evaluated the expression of apoptosis-related proteins by Western blot analysis (Figure 5F). Treatment of AZD1775 strongly inhibited WEE1 and Cdc2-phosphorylation, and increased cleaved caspase-3 and cleaved PARP. In addition, AZD1775 treatment led to an increase in γ-H2AX expression. The data indicated that AZD1775 might also induce DNA damage. Interestingly, the expression level of Cyclin B1 showed the difference between p53 mutation statuses in these cells. Cyclin B1 was increased at 24 hr in all four cell lines, but rapidly decreased after 48 hr of AZD1775 treatment in p53 wild type AGS and YCC-2 cells. However, increased Cyclin B1 expression at 24 hrs was sustained up to 48 hrs in p53 mutant MKN28 and SNU-601 cells, suggesting that these cells were under cell cycle arrest at the G2/M phase [22]. This result was also corresponded with our cell cycle data, Figure 5A.

### Combined treatment with AZD1775 and anti-cancer agents, 5-FU and Paclitaxel, enhanced the therapeutic effects on gastric cancer cells

**Figure 6:**
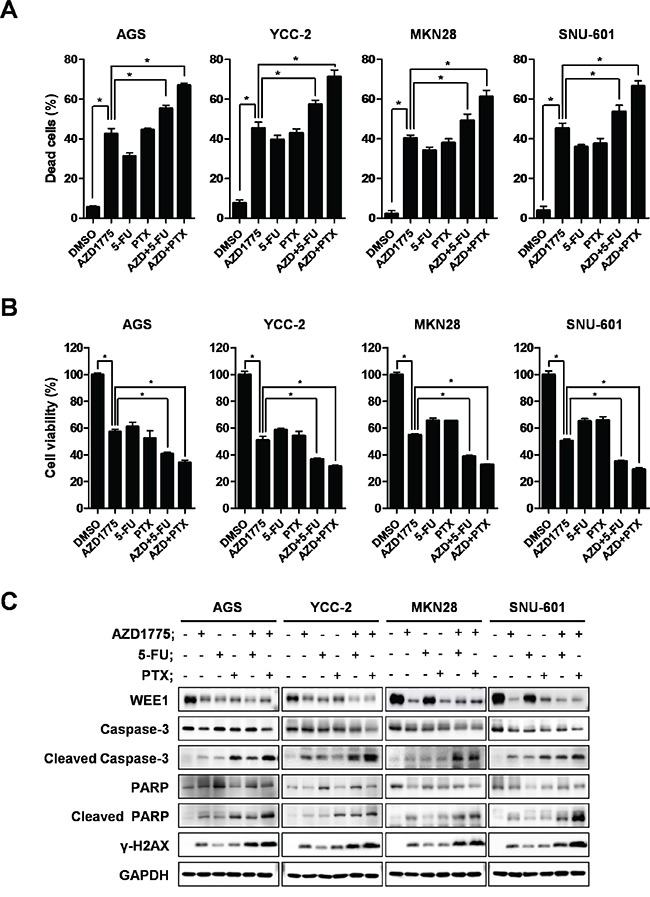
Apoptosis induction is detected after combination treatment with AZD1775, 5-FU, and paclitaxel Apoptosis of AGS, YCC-2, MKN28, and SNU-601 cells were detected by FACS analysis using annexin-V staining. **A.** Quantitative data are presented graphically. WST assays were performed to detect cell viability. **B.** Data is represented as mean ± SD (n=3). Significant differences are indicated by asterisk (* p<0.05), p values calculated using ANOVA. **C.** Apoptosis related proteins were detected by Western blot. AGS, YCC-2, MKN28, and SNU-601 cells were treated with single agents or combination therapy for 48 hrs. GAPDH was used as a loading control. 5-FU: 5-Fluorouracil; PTX: paclitaxel; AZD+5-FU: AZD1775 and 5-FU combination treatment; AZD+PTX: AZD1775 and paclitaxel combination treatment.

To determine the practical applications of AZD1775 in gastric cancer therapy, we conducted a combination treatment with AZD1775, 5- fluorouracil (5-FU) and paclitaxel (PTX) in gastric cancer cells. Gastric cancer cells were treated with AZD1775 alone (0.5 μM of AZD1775 in AGS and YCC-2 cells, and 1μM of AZD1775 in MKN28 and SNU-601 cells), 5-FU alone (0.5μg/ml), PTX alone (0.2μM) or a combination of AZD1775 and 5-FU or AZD1775 and PTX (each at half concentration). We investigated cell viability and apoptosis induction after combination treatments. After 48 hrs, we performed WST assays for cell viability and each combination treatment showed greater inhibition of cell viability than treatment with the single agents alone (Figure 6A). Combination treatments also increased apoptosis induction more than the single treatments in gastric cancer cells (Figure 6B and Figure S3). Apoptosis induction markers, caspase-3 and PARP, were cleaved after combination treatments and DNA damage marker, γ-H2AX, was also greatly induced by combination treatment (Figure 6C). These data suggest that AZD1775 enhanced the therapeutic effects on gastric cancer when it was used in combination treatments with 5-FU or PTX.

### Combined treatment with AZD1775 and anti-cancer agents, 5-FU and Paclitaxel, enhanced the therapeutic effect on orthotopic transplanted gastric cancer mice

**Figure 7:**
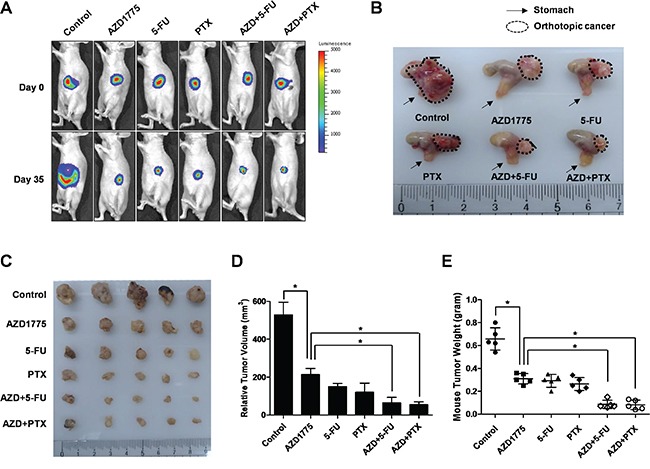
The effect of AZD1775 and anti-cancer agent combination treatment on the orthotopic mouse model for gastric cancer **A.** Monitoring luciferase inhibition *in vivo* with bioluminescent imaging. Mice were given 100 μl of the control, 20mg/kg/2days AZD1775, 10mg/kg/2days 5-FU, and 5mg/kg/2days Paclitaxel, or a combination of 20mg/kg/2days AZD1775 and 10mg/kg/2days 5-FU, or a combination of 20mg/kg/2days AZD1775 and 5mg/kg/2days Paclitaxel by oral gavage (AZD1775) or intraperitoneal injections (5-FU and Paclitaxel). **B.** Mice were sacrificed and the orthotopic gastric tumor was obtained. Arrow is mouse stomach and dotted line is orthotopic cancer. **C-E.** Photographs and quantification of tumor formation was performed by measuring tumor size and weight 35 days after chemotherapy. Significance differences are indicated by asterisk (* p<0.05), p-values calculated using ANOVA.

We established a mouse model of orthotopic human gastric cancer, which closely mimics the physiology of human gastric cancers. AGS-luciferase gastric cancer cells containing the luciferase gene as an indicator were surgically transplanted into the epithelia of mouse stomachs. We confirmed the therapeutic effects of the combination of AZD1775 and anti-cancer agents, 5-FU and Paclitaxel, using our *in vivo* mouse model. Four weeks after transplantation, we administered control single treatments (AZD1775, 5-FU, and PTX), or a combination of AZD1775 with 5-FU and AZD1775 with Paclitaxel by oral gavage (AZD1775) or intraperitoneal injection(5-FU and PTX). Gastric cancer orthotopic mouse tumor growth was measured by tomographic imaging (Figure 7A). We also analyzed the toxic side effects of AZD1775 and anti-cancer agents in these mice. There was no weight loss in the mice that received AZD1775 and the anti-cancer agents (Figure S4A). Nine weeks after transplantation, there was a suppression of tumor growth in mice treated AZD1775 and those undergoing combination therapy (Figure 7B). After isolating the tumors from the mouse stomachs, their size and weight were calculated (Figure 7C-7E). The tumor size and weight of AZD1775 treated mice were reduced compared to the control mice (Figure 7C-7E). In addition, tumor size and weight of mice undergoing combination therapy with AZD1775 were also decreased (Figure 7C-7E). These studies demonstrate thatAZD1775 treatment alone is effective in suppressing gastric cancer. Also, combination treatment induced suppression of the growth of gastric cancers in the mouse model as compared with single-drug treatment.

## DISCUSSION

Previously, it has been reported that WEE1 is highly expressed in several cancers and has oncogenic roles [20]. However, it is not well studied in gastric cancers. In this study, we determined for the first time the association between WEE1 expression and survival probability using clinical data from gastric cancer patients as shown in Figure 1. We show that high-expression of WEE1 at stage 4 showed a statistically significant poor survival rate compared to the expression level of early stage gastric cancer patients. Interestingly, male WEE1 high-expression patients had poorer survival rates than male WEE1 low-expression patients. Furthermore, male gastric cancer patients with advanced lymph node metastases had high expression of WEE1 and were associated with poor survival probability. Therefore, we further investigated *in vitro* and *in vivo* whether targeting WEE1 has therapeutic potential in gastric cancer. The functional impact of WEE1 after silencing or over-expression on cell viability, invasion, and migration was investigated. Inhibition of WEE1 led to decreased cell viability, invasion, and migration of WEE1 high-expressed gastric cancer cells, whereas WEE1 overexpression reversed these effects in WEE1 low-expressed gastric cancer cells. This suggests that WEE1 regulates the cell proliferation and motility of gastric cancer cells. We focused on the advantage of inhibiting WEE1 and how that may inhibit the epithelial-mesenchymal transition (EMT), which is associated with the metastasis of cancer [23]. There was a correlation with highly expressed WEE1, which showed poor survival probability in lymph node metastasized male patients. However, ithas not been reported how WEE1 regulates cell motility and metastasis and we are currently working to find the molecular mechanism.

We also suggest that AZD1775, a WEE1 inhibitor, could be a potent anti-gastric cancer agent that could be applied in clinical trials. AZD1775 has been reported for its anti-cancer effects in various cancer cells [12, 15, 24, 25] and is undergoing clinical trials [17]. In this study, we showed the effect of the WEE1 inhibitor, AZD1775, on gastric cancer cells. Treatment of AZD1775 in AGS and YCC-2 cells (p53 wild type) induced G1/S phase arrest, where as treatment of AZD1775 in MKN28 (p53 I251L mutant) and SNU-601 cells (p53 R273H mutant) induced G2/M cell cycle arrest). It was confirmed that the killing sensitivity of AZD1775 is higher in p53 wild type gastric cancer cells. We used IC_50_ dose to detect cell cycle and apoptosis. IC_50_ of AZD1775 is 0.5 μM in p53 wild type gastric cancer cells (AGS and YCC2 cells), and 1 μM in p53 mutant gastric cancer cells (MKN28 and SNU-601 cells). However, it was interesting that IC_50_ of AZD1775 induced cell-cycle arrest differently, between p53 wild type and mutant cells. AZD1775 induced G1 arrest in p53 wild type cells and G2/M arrest in p53 mutant type cells. p53 is the G1/S checkpoint and a master regulator for DNA damage responses in the S phase [4]. p53 mutant cancer cells are therefore dependent on the intra-S phase and G2/M checkpoints [26]. It has been suggested that AZD1775 causes DNA damage in both p53 wild type and mutant cells, and this is supported by the increased expression level of γ-H2AX in both type of cells [27]. Induced DNA damage by AZD1775 increased the sub-G1 population through S phase arrest in p53 wild type gastric cancer cells and also increased the sub-G1 population through G2/M phase arrest in p53 mutant gastric cancer cells. We suggested here that the difference between G1 arrest and G2/M arrest causes the different killing sensitivity of AZD1775.

In previous studies, AZD1775 has been shown to have a synergistic effect in DNA damage–based therapeutics by inducing unscheduled mitosis and eventually resulting in apoptosis in various cancers, such as melanoma, glioblastoma, and pancreatic cancer [28–30]. We also assessed the clinical potential of AZD1775 when combined with anti-cancer drugs, such as 5-FU (a DNA damage agent) and Paclitaxel (a mitotic inhibitor). Because AZD1775 induces DNA damage in cells [19], we hypothesized that enhanced DNA damage by combination treatment with AZD1775 and 5-FU/paclitaxel in cancer cells is more effective than single agent treatment. 5-Fluorouracil (5-FU) is a pyrimidine analog which is used in the treatment of cancer. It works through irreversible inhibition of thymidylate synthase. 5-FU is about widely used in cancer therapy. Paclitaxel (Taxol) is an anti-cancer chemotherapy drug and one of several cytoskeletal drugs. Treated paclitaxel in cells have defects in mitotic spindle assembly and cell division. And also induce DNA single-strand breaks (DSB) in some tumor cell [31]. However, those anti-cancer agents have side effects when used excessively. Therefore, we also expected the reduced toxicity by these combination treatments. Moreover, we also established a gastric tumor orthotopic transplanted mouse model for more therapeutic accuracy [22, 32] and administrated the combination treatment in these mice. Even though 5-FU and paclitaxel have been previously shown to have significant anti-cancer effects in gastric cancer, the combination treatment showed higher efficiency to diminish the gastric tumor burden than the single agent treatments.

Taken together, we demonstrate that high expression of WEE1 in advanced stages and/or accompanied with lymph node metastasis indicate poorer survival for gastric cancer patients and that targeting WEE1 would be a therapeutic benefit for gastric cancer patients.

## MATERIALS AND METHODS

### Cell culture

Twelve human gastric cancer cell lines (AGS, YCC-2, MKN28, KATO III, SNU-1, SNU-5, SNU-16, SNU-216, SNU-601, SNU-638, SNU-668, and SNU-719) obtained from the Korea Cell Line Bank (KCLB, Korea) were cultured in RPMI-1640 medium supplemented with 10% fetal bovine serum (FBS: Corning Costar, USA) and 1% antibiotic-antimycotic (Gibco, USA). Cell cultures were maintained at 37°C in an atmosphere of 5% CO_2_. The phenotypes of these cell lines have been authenticated by the KCLB.

### siRNA transfection and WEE1 over-expression construction

Scramble RNA (scRNA) and WEE1 siRNA transfections were performed with the LipofectamineRNAiMAX reagent (Invitrogen, USA), according to the manufacturer's instructions as previously described [33, 34]. The primer sequences were as follows: scRNA: 5′-UUCUCCGAACGUGUCACGU-3′;human WEE1 siRNA: 5′-GGCUGGAUGGAUGCA UUUAUU-3′. WEE1 siRNA was purchased from Genolution (Korea). The empty vector (pcDNA3.0/FLAG) and WEE1 over-expression vector (pcDNA3.0/FLAG-WEE1) were transiently transfected into cells using the Lipofectamine 2000 reagent (Invitrogen), according to the manufacturer's instructions.

### Transwell migration and invasion assays

AGS, YCC-2, MKN28 and SNU-601 cells were transfected with scRNA and WEE1 siRNA. KATO III and SNU-668 were transfected with empty vectors and the WEE1 over-expression vector. After transfection for 24 hr, cells (AGS-1 × 10^4^, YCC-2-1 × 10^4^, MKN28-1 × 10^4^, SNU-601-1 × 10^4^, KATO III-1 × 10^4^, and SNU-668-1 × 10^4^ in each well) were isolated and added to the upper Transwell (Corning Costar, USA) chambers with 0.5 mg/ml collagen type I (BD bioscience, Korea)-coated filters for the migration assay, and with a 1/15 dilution of Matrigel (BD bioscience, Korea)-coated filters for the invasion assays. RPMI 1640 containing 10% FBS and 1% antibiotics was added to the lower chamber and incubation was continued for 20 hrs. Cells that migrated or invaded the lower chamber were quantified after H&E staining as previously described [35–37]. For quantification, cells were counted in 5 randomly selected areas in each well using wide-field microscopy. Data were expressed as mean ± SD from three independent experiments.

### Cell proliferation detection assays

AGS, YCC-2, MKN28, SNU-601, KATO III, and SNU-668 cells were plated in 96-well culture plates (3 × 10^3^ per well). After incubation for 24 hr, AGS, YCC-2, MKN28 and SNU-601 cells were transfected with scRNA and WEE1 siRNA. KATO III and SNU-668 cells were transfected with an empty vector and the WEE1 over-expression vector. After transfection for 48 hr, WST solution (Daeil, korea) was subsequently added to each well. After 1 hr of additional incubation, the plate was shaken gently. The absorbance was measured on an ELISA reader at a test wavelength of 450 nm as previously described [38]. Inhibition of cell proliferation by AZD1775 alone or in combination with 5-FU or Paclitaxel was measured using the WST assay. The WEE1 inhibitor, AZD1775, was purchased from Selleckchem (Houston, TX, USA) and dissolved in dimethyl sulfoxide (DMSO). The AGS, YCC-2, MKN28, and SNU-601 cells were plated in 96-well culture plates (3 × 10^3^ per well). After incubation for 24 hr, the cells were treated with AZD1775 for 48 hrs. WST solution (Daeil) was subsequently added to each well. After 1 hr of additional incubation, the plate was shaken gently. The absorbance was measured on an ELISA reader at a test wavelength of 450 nm.

### Crystal violet staining assay

AGS, YCC-2, MKN28, and SNU-601 cells were plated in 6well culture plates and treated with AZD1775 (0.5 μM or 1 μM) for 48 hr. Washing the cells with 1X PBS and fixing by 10 min exposure to 1% glutaraldehyde (Sigma). After fixation, washing with 1X PBS. Stain cells with 0.5% Crystal violet (Sigma) for 10 min at RT.

### Cell cycle analysis

AGS, YCC-2, MKN28, and SNU-601 cells were plated in culture plates and treated with AZD1775 (0.5 μM or 1 μM) for time-dependent course (24, 48, and 72 hr). Cells were harvested and washed twice with cold PBS, and then resuspended cells in 5ml 70% EtOH overnight at −20 °C. After fixation, the cells were washed twice with cold PBS and resuspended in Propidium Iodide Staining solution (PI solution; RNaseA 50 μg/ml, PI 50 μg/ml in PBS) and transferred to FACS filter tubes. Cell cycle distribution after AZD1775 treatment (AGS-0.5 μM, YCC-2-0.5 μM, MKN28-1μM, and SNU-601-1μM in each well) was measured by PI staining using fluorescence-activated cell sorting (FACS) [39].

### Apoptosis detection assays

AGS, YCC-2, MKN28, and SNU-601 cells were plated onto culture plates and treated with AZD1775 only (AGS-0.5 μM, YCC-2-0.5 μM, MKN28-1 μM, and SNU-601-1 μM each well), 5-Fluorouracil (5-FU) only (0.5 μg/ml) or a combination of AZD1775 (AGS-0.5 μM, YCC-2-0.5 μM, MKN28-1 μM, and SNU-601-1 μM in each well) and Paclitaxel (PTX; 0.2 μM) for 48 hr. After the time passed, cells were harvested. Cells were washed twice with cold PBS and then resuspended in1X Annexin V Binding at a concentration of 1 × 10^6^ cells/ml. Then, 100 μl of the solution (1 × 10^5^ cells) was transferred to a 1 ml culture tube and 5 μl of PE Annexin V and 5 μl 7-AAD each sample. The cells were gently vortexed and incubated for 15 min at RT in the dark. We added 400 μl of 1X Annexin Binding Buffer to each tube and transferred the solution to FACS filter tubes. Apoptosis distribution after AZD1775 with 5-FU or Paclitaxel treatment was measured by Annexin V staining using FACS [40].

### Western blotting

Cell lysate extractions were prepared with RIPA buffer 1% NP-40; 0.1% sodium dodecyl sulfate; 0.5% desoxycholate; 150 mM NaCl; 50 mM Tris, pH 7.5) and a protease inhibiter cocktail. 20 μg total protein of each lysate was resolved in SDS PAGE gels and electro-transferred to PVDF membranes, and then blocked in 5% skim milk in 0.05% Tween-20 with 1X PBS (PBST). Primary antibodies were incubated with the blots at a 1:1000 dilution in minimal volumes of 5% BSA (Bovine serum albumin) in PBST buffer for 1 hr at room temperature or over-night at 4 °C. Anti-mouse or anti-rabbit goat-HRP-conjugated secondary antibodies were incubated at a 1:5000 dilution in 5% BSA in PBST buffer for 1.5 hrs at room temperature. Antibodies used in this study were anti-WEE1, anti-Cdc2 p34, anti-phospho-Cdc2 p34 (Thr 14/Tyr15), anti-Cyclin B1, and anti-GAPDH that were purchased from Santa Cruz Biotechnology. Anti-caspase-3 and anti-PARP were obtained from Cell Signaling. Anti-phospho-histone H2A.X was purchased from Milipore Corpoation. Anti-mouse and anti-rabbit polyclonal immunoglobulins were purchased from Bethyl Laboratories. Membranes that were probed with primary antibodies and secondary antibodies were detected by ECL solution (Amersham Life Science) using a LAS-3000 (Fujifilm) detector, according to the manufacturer's directions.

### Immunocytochemistry and confocal microscopy

Cells were cultured in chamber slide and fixed with 3.7% formaldehyde, following by permeabilization with 0.5% Triton X-100. The cells were blocked with 5% BSA in PBS and then incubated with primary anti-phospho-Histon H3 (Ser 10), anti-phospho-histone H2A.X diluted (1:200) in PBS. The cells were conjugated with the secondary antibodies labeled with FITC diluted (1:200) in PBS. The samples were treated with mounting medium with DAPI. The chamber slide was covered with cover glass and analyzed on a confocal microscope (Carl Zeiss).

### Statistical analysis

Significant differences between the treatment and control groups were determined using the paired *t*test and ANOVA for multiple samples (indicated). Differences were considered significant if the *P* value was less than 0.05. Analysis of data was done using the Prism 5 software.

### Kaplan–Meier analysis of relapse-free survival

Kaplan–Meier analysis of the survival curve was generated using the online resource (http://kmplot.com/) analysis and gene set for gastric cancer patients as previously described [41].

### Preparation of orthotopically transplanted gastric cancer bearing mouse models

All animal experiments were approved by the Institutional Review Board of the Yonsei University College of Medicine and were performed in specific pathogen-free facilities, in accordance with the University's Guidelines for the Care and Use of Laboratory Animals (2015-0087). Six-week old female Balb/c-nude mice (Orient, Korea) were subcutaneously inoculated with AGS luciferase cells (1 × 10^6^) in the side, as previously reported [42–45]. **S**ubcutaneous tumors were excised and implanted into the gastric wall of nude mice. Mice were randomized into groups (n=5 per group) and treatment was started 4 weeks after tumor implantation. Mice received 100 μl of controls, 20mg/kg/2days AZD1775, 10mg/kg/2days 5-FU, 5mg/kg/2days Paclitaxel, a combination of 20mg/kg/2days AZD1775 and 10mg/kg/2days 5-FU, or a combination of 20mg/kg/2days AD1775 and 5mg/kg/2days Paclitaxel by oral gavage (AZD1775) or intraperitoneal (i.p.) injection(5-FU and Paclitaxel). Treatments were given three times/week for 5weeks. Once every two weeks, the mice were injected i.p. with luciferin (Xenogen, Alameda, CA) and luciferase activity was measured by IVIS imaging. The experiment was terminated at 5 weeks and the orthotopically placed tumors were calculated using the formula *a*^2^ × *b* × 0.5.

## SUPPLEMENTARY MATERIALS FIGURES



## References

[1] McLean MH, El-Omar EM (2014). Genetics of gastric cancer. Nature reviews Gastroenterology & hepatology.

[2] Riquelme I, Saavedra K, Espinoza JA, Weber H, Garcia P, Nervi B, Garrido M, Corvalan AH, Roa JC, Bizama C (2015). Molecular classification of gastric cancer: Towards a pathway-driven targeted therapy. Oncotarget.

[3] Khanna A (2015). DNA damage in cancer therapeutics: a boon or a curse?. Cancer research.

[4] Dixon H, Norbury CJ (2002). Therapeutic exploitation of checkpoint defects in cancer cells lacking p53 function. Cell cycle.

[5] De Witt Hamer PC, Mir SE, Noske D, Van Noorden CJ, Wurdinger T (2011). WEE1 kinase targeting combined with DNA-damaging cancer therapy catalyzes mitotic catastrophe. Clinical cancer research.

[6] Featherstone C, Russell P (1991). Fission yeast p107wee1 mitotic inhibitor is a tyrosine/serine kinase. Nature.

[7] McGowan CH, Russell P (1995). Cell cycle regulation of human WEE1. The EMBO journal.

[8] Russell P, Nurse P (1987). Negative regulation of mitosis by wee1+, a gene encoding a protein kinase homolog. Cell.

[9] Gould KL, Nurse P (1989). Tyrosine phosphorylation of the fission yeast cdc2+ protein kinase regulates entry into mitosis. Nature.

[10] Sanchez V, McElroy AK, Spector DH (2003). Mechanisms governing maintenance of Cdk1/cyclin B1 kinase activity in cells infected with human cytomegalovirus. Journal of virology.

[11] Mahajan K, Fang B, Koomen JM, Mahajan NP (2012). H2B Tyr37 phosphorylation suppresses expression of replication-dependent core histone genes. Nature structural & molecular biology.

[12] Mir SE, De Witt Hamer PC, Krawczyk PM, Balaj L, Claes A, Niers JM, Van Tilborg AA, Zwinderman AH, Geerts D, Kaspers GJ, Peter Vandertop W, Cloos J, Tannous BA, Wesseling P, Aten JA, Noske DP (2010). In silico analysis of kinase expression identifies WEE1 as a gatekeeper against mitotic catastrophe in glioblastoma. Cancer cell.

[13] Magnussen GI, Holm R, Emilsen E, Rosnes AK, Slipicevic A, Florenes VA (2012). High expression of Wee1 is associated with poor disease-free survival in malignant melanoma: potential for targeted therapy. PloS one.

[14] Murrow LM, Garimella SV, Jones TL, Caplen NJ, Lipkowitz S (2010). Identification of WEE1 as a potential molecular target in cancer cells by RNAi screening of the human tyrosine kinome. Breast cancer research and treatment.

[15] Kreahling JM, Foroutan P, Reed D, Martinez G, Razabdouski T, Bui MM, Raghavan M, Letson D, Gillies RJ, Altiok S (2013). Wee1 inhibition by MK-1775 leads to tumor inhibition and enhances efficacy of gemcitabine in human sarcomas. PloS one.

[16] Mueller S, Hashizume R, Yang X, Kolkowitz I, Olow AK, Phillips J, Smirnov I, Tom MW, Prados MD, James CD, Berger MS, Gupta N, Haas-Kogan DA (2014). Targeting Wee1 for the treatment of pediatric high-grade gliomas. Neuro-oncology.

[17] Do K, Wilsker D, Ji J, Zlott J, Freshwater T, Kinders RJ, Collins J, Chen AP, Doroshow JH, Kummar S (2015). Phase I Study of Single-Agent AZD1775 (MK-1775), a Wee1 Kinase Inhibitor, in Patients With Refractory Solid Tumors. Journal of clinical oncology.

[18] Mizuarai S, Yamanaka K, Itadani H, Arai T, Nishibata T, Hirai H, Kotani H (2009). Discovery of gene expression-based pharmacodynamic biomarker for a p53 context-specific anti-tumor drug Wee1 inhibitor. Molecular cancer.

[19] Hirai H, Arai T, Okada M, Nishibata T, Kobayashi M, Sakai N, Imagaki K, Ohtani J, Sakai T, Yoshizumi T, Mizuarai S, Iwasawa Y, Kotani H (2010). MK-1775, a small molecule Wee1 inhibitor, enhances anti-tumor efficacy of various DNA-damaging agents, including 5-fluorouracil. Cancer biology & therapy.

[20] Do K, Doroshow JH, Kummar S (2013). Wee1 kinase as a target for cancer therapy. Cell cycle.

[21] Chaudhuri L, Vincelette ND, Koh BD, Naylor RM, Flatten KS, Peterson KL, McNally A, Gojo I, Karp JE, Mesa RA, Sproat LO, Bogenberger JM, Kaufmann SH, Tibes R (2014). CHK1 and WEE1 inhibition combine synergistically to enhance therapeutic efficacy in acute myeloid leukemia ex vivo. Haematologica.

[22] Kim SJ, Lee HW, Baek JH, Cho YH, Kang HG, Jeong JS, Song J, Park HS, Chun KH (2016). Activation of nuclear PTEN by inhibition of Notch signaling induces G2/M cell cycle arrest in gastric cancer. Oncogene.

[23] Palmer TD, Ashby WJ, Lewis JD, Zijlstra A (2011). Targeting tumor cell motility to prevent metastasis. Advanced drug delivery reviews.

[24] Bridges KA, Hirai H, Buser CA, Brooks C, Liu H, Buchholz TA, Molkentine JM, Mason KA, Meyn RE (2011). MK-1775, a novel Wee1 kinase inhibitor, radiosensitizes p53-defective human tumor cells. Clinical cancer research.

[25] Guertin AD, Li J, Liu Y, Hurd MS, Schuller AG, Long B, Hirsch HA, Feldman I, Benita Y, Toniatti C, Zawel L, Fawell SE, Gilliland DG, Shumway SD (2013). Preclinical evaluation of the WEE1 inhibitor MK-1775 as single-agent anticancer therapy. Molecular cancer therapeutics.

[26] Aarts M, Linardopoulos S, Turner NC (2013). Tumour selective targeting of cell cycle kinases for cancer treatment. Current opinion in pharmacology.

[27] Kiviharju-af Hallstrom TM, Jaamaa S, Monkkonen M, Peltonen K, Andersson LC, Medema RH, Peehl DM, Laiho M (2007). Human prostate epithelium lacks Wee1A-mediated DNA damage-induced checkpoint enforcement. Proceedings of the National Academy of Sciences of the United States of America.

[28] Magnussen GI, Emilsen E, Giller Fleten K, Engesaeter B, Nahse-Kumpf V, Fjaer R, Slipicevic A, Florenes VA (2015). Combined inhibition of the cell cycle related proteins Wee1 and Chk1/2 induces synergistic anti-cancer effect in melanoma. BMC cancer.

[29] Pokorny JL, Calligaris D, Gupta SK, Iyekegbe DO, Mueller D, Bakken KK, Carlson BL, Schroeder MA, Evans DL, Lou Z, Decker PA, Eckel-Passow JE, Pucci V, Ma B, Shumway SD, Elmquist WF (2015). The Efficacy of the Wee1 Inhibitor MK-1775 Combined with Temozolomide Is Limited by Heterogeneous Distribution across the Blood-Brain Barrier in Glioblastoma. Clinical cancer research.

[30] Wang G, Niu X, Zhang W, Caldwell JT, Edwards H, Chen W, Taub JW, Zhao L, Ge Y (2015). Synergistic antitumor interactions between MK-1775 and panobinostat in preclinical models of pancreatic cancer. Cancer letters.

[31] Branham MT, Nadin SB, Vargas-Roig LM, Ciocca DR (2004). DNA damage induced by paclitaxel and DNA repair capability of peripheral blood lymphocytes as evaluated by the alkaline comet assay. Mutation research.

[32] Lee HW, Kim SJ, Choi IJ, Song J, Chun KH (2015). Targeting Notch signaling by gamma-secretase inhibitor I enhances the cytotoxic effect of 5-FU in gastric cancer. Clinical & experimental metastasis.

[33] Cho Y, Lee HW, Kang HG, Kim HY, Kim SJ, Chun KH (2015). Cleaved CD44 intracellular domain supports activation of stemness factors and promotes tumorigenesis of breast cancer. Oncotarget.

[34] Choi SW, Song JK, Yim YS, Yun HG, Chun KH (2015). Glucose deprivation triggers protein kinase C-dependent beta-catenin proteasomal degradation. The Journal of biological chemistry.

[35] Kim SJ, Choi IJ, Cheong TC, Lee SJ, Lotan R, Park SH, Chun KH (2010). Galectin-3 increases gastric cancer cell motility by up-regulating fascin-1 expression. Gastroenterology.

[36] Kim SJ, Wang YG, Lee HW, Kang HG, La SH, Choi IJ, Irimura T, Ro JY, Bresalier RS, Chun KH (2014). Up-regulation of neogenin-1 increases cell proliferation and motility in gastric cancer. Oncotarget.

[37] Wang YG, Kim SJ, Baek JH, Lee HW, Jeong SY, Chun KH (2012). Galectin-3 increases the motility of mouse melanoma cells by regulating matrix metalloproteinase-1 expression. Experimental & molecular medicine.

[38] Lee HW, Jang KS, Choi HJ, Jo A, Cheong JH, Chun KH (2014). Celastrol inhibits gastric cancer growth by induction of apoptosis and autophagy. BMB reports.

[39] Mi Y, Zhang C, Bu Y, Zhang Y, He L, Li H, Zhu H, Li Y, Lei Y, Zhu J (2015). DEPDC1 is a novel cell cycle related gene that regulates mitotic progression. BMB reports.

[40] Kim GY, Park SY, Jo A, Kim M, Leem SH, Jun WJ, Shim SI, Lee SC, Chung JW (2015). Gecko proteins induce the apoptosis of bladder cancer 5637 cells by inhibiting Akt and activating the intrinsic caspase cascade. BMB reports.

[41] Kim SJ, Hwang JA, Ro JY, Lee YS, Chun KH (2013). Galectin-7 is epigenetically-regulated tumor suppressor in gastric cancer. Oncotarget.

[42] Kim SJ, Oh JS, Shin JY, Lee KD, Sung KW, Nam SJ, Chun KH (2011). Development of microRNA-145 for therapeutic application in breast cancer. J Control Release.

[43] Ko A, Shin JY, Seo J, Lee KD, Lee EW, Lee MS, Lee HW, Choi IJ, Jeong JS, Chun KH, Song J (2012). Acceleration of gastric tumorigenesis through MKRN1-mediated posttranslational regulation of p14ARF. Journal of the National Cancer Institute.

[44] Lee EW, Kim JH, Ahn YH, Seo J, Ko A, Jeong M, Kim SJ, Ro JY, Park KM, Lee HW, Park EJ, Chun KH, Song J (2012). Ubiquitination and degradation of the FADD adaptor protein regulate death receptor-mediated apoptosis and necroptosis. Nature communications.

[45] Ahn YH, Yi H, Shin JY, Lee KD, Shin SP, Lee SJ, Song J, Chun KH (2012). STAT3 silencing enhances the efficacy of the HSV. tk suicide gene in gastrointestinal cancer therapy. Clinical & experimental metastasis.

