# Supplementary material for: Targeting the WEE1 kinase as a molecular targeted therapy for gastric cancer
Source: Oncotarget. 2016 Jun 23;7(31):49902–16. doi: 10.18632/oncotarget.10231 (PMC5226556; doi:10.18632/oncotarget.10231)
Supplement: Supplementary file 1 [file oncotarget-07-49902-s001.pdf]

Targeting the WEE1 kinase as a molecular targeted therapy for gastric cancer

SUPPLEMENTARY FIGURES

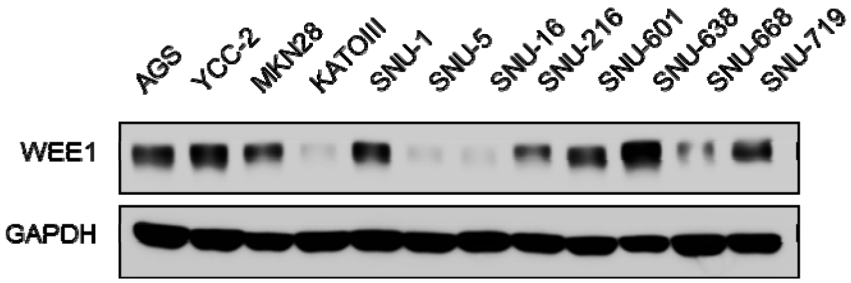

Supplementary Figure S1: Basal level of WEE1 expression in twelve human gastric cancer cell lines. Protein expression levels of WEE1 were detected by western blot in indicated gastric cancer cell lines. GAPDH was used as loading control.

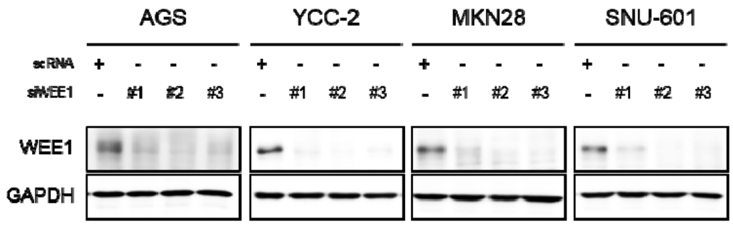

	5′→3′ sequence
scRNA	UUCUCCGAACGUGUCACGU
siWEE1 #1	GGCUGGAUGGAUGCAUUUAUU
siWEE1 #2	GCCAGUGAUUAUGAGCUUGAA
siWEE1 #3	CCACCCAGAGUAAUAGAAUAU

Supplementary Figure S2: Used three different sequences of siRNA in this study. Three different sequences of siRNA (described in below table) were prepared and checked WEE1 knockdown efficiency in four gastric cancer cell lines (AGS, YCC-2, MKN28 and SNU-601) by western blot analysis.

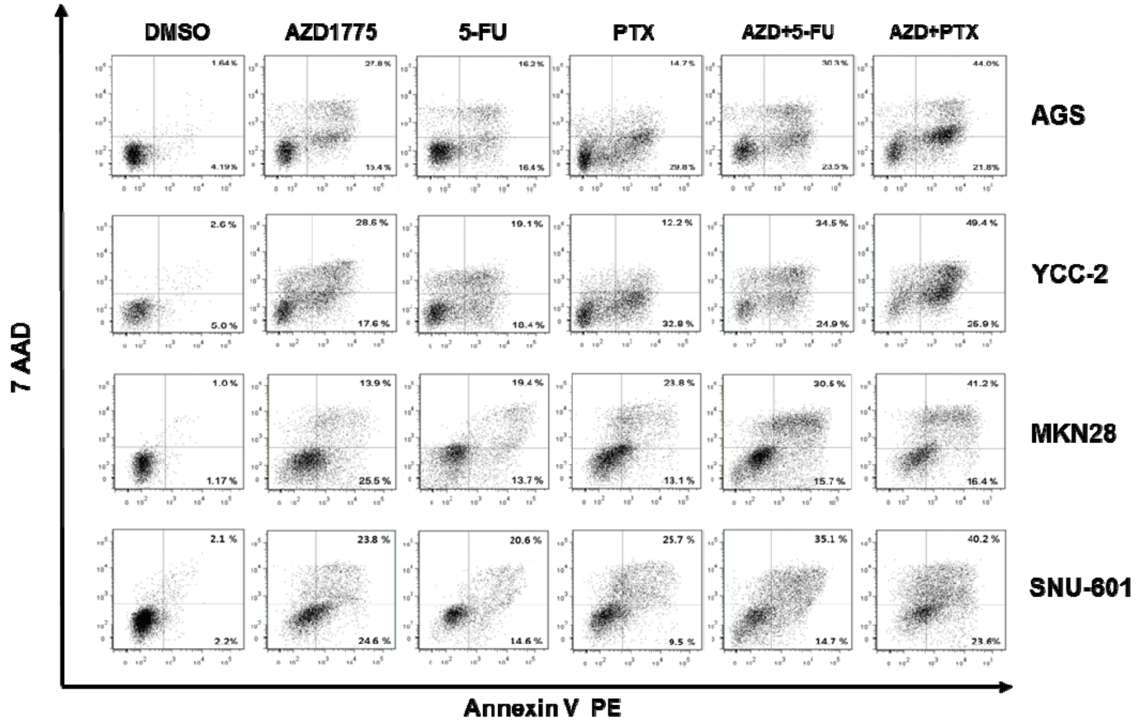

Supplementary Figure S3: Combination therapy leads to apoptotic cell death. Apoptosis induction of AGS, YCC-2, MKN28 and SNU-601 cells were detected by FACS analysis using an annexin-V staining.

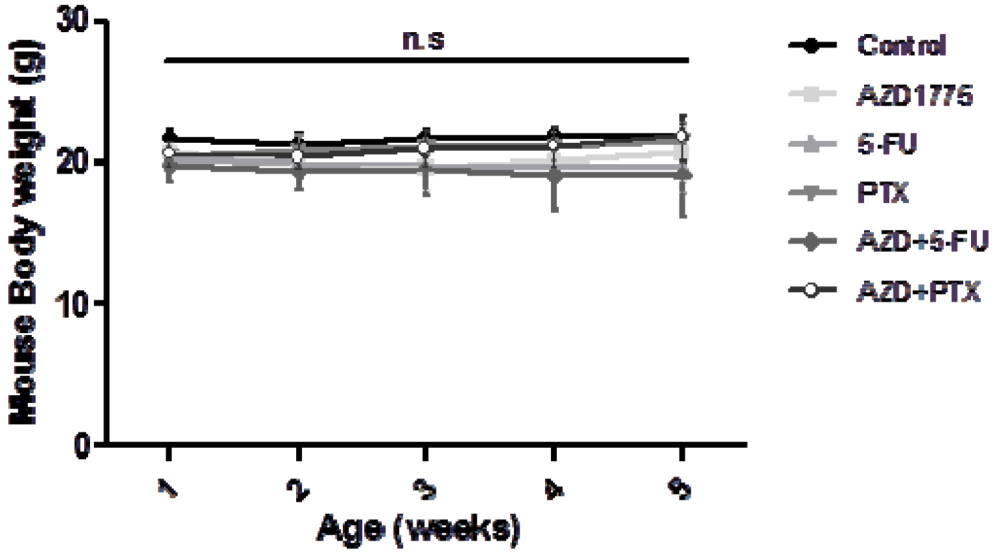

Supplementary Figure S4: Combination treatment on orthotopic mouse models of gastric cancer. The graph for Body weight curves of control, AZD1775, 5-FU, PTX, AZD+5-FU and AZD+PTX (n=5) mice.
